# Spatial-Semantic Object Relation Graph Networks for Vehicle Attachment Detection in Automatic Car Wash System

**DOI:** 10.3390/s26082464

**Published:** 2026-04-16

**Authors:** Hyeongseop Lim, Changwoo Nam, Sang Jun Lee

**Affiliations:** Division of Electronic Engineering, Jeonbuk National University, 567 Baekje-daero, Deokjin-gu, Jeonju 54896, Republic of Korea; hungsub1234@jbnu.ac.kr (H.L.); cw.nam@jbnu.ac.kr (C.N.)

**Keywords:** car washing machine, vehicle attachment detection, computer vision, deep learning, graph neural network

## Abstract

Precise object detection is critical for preventing damage to vehicle attachments during automatic car washing. However, the existing methods often suffer from low accuracy and false detections due to the diverse shapes and visual ambiguity of these attachments. To address these challenges, we propose a novel framework integrating a YOLOv11-based detector with a graph neural network. Specifically, we introduce a spatial graph module to refine object localization by capturing invariant spatial constraints within the car wash environment. Furthermore, we incorporate a class graph module to model inter-class semantic correlations, thereby improving the classification of visually ambiguous objects such as emblems. Experimental results on a real-world dataset demonstrate that our method achieves an mAP50 of 97.9%, outperforming state-of-the-art models including D-FINE 96.5% and RT-DETR 96.1%. These findings confirm the robustness of our approach under varying viewpoints and background conditions, offering a significant improvement in the safety and reliability of automatic car wash systems.

## 1. Introduction

**Figure 1:**
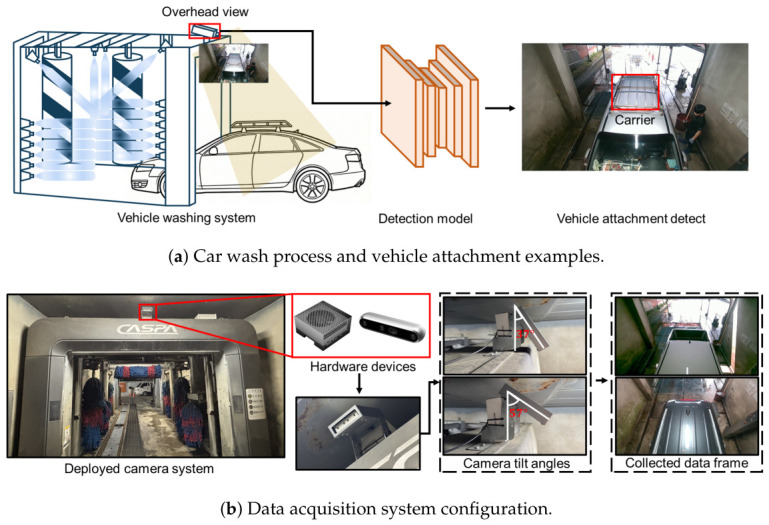
Overview of the automatic car wash system and data acquisition setup. (**a**) Car wash process illustrating vehicle attachments prone to damage during washing. (**b**) Data acquisition system deployed at the car wash entrance for dataset collection.

Driven by the increasing number of registered vehicles and rising labor costs, the vehicle cleaning industry is transitioning from traditional manual methods to efficient automatic car wash systems [1]. These systems typically employ high-pressure water jets and high-speed rotating brushes to remove surface contaminants [2,3,4]. While this automation enhances throughput and cleaning consistency, it introduces a significant risk of damaging protruding exterior attachments due to the physical forces involved. As illustrated in Figure 1a, vehicles entering the car wash are frequently equipped with various exterior attachments, such as emblems, antennas, and carriers, which must be accurately identified by the detection system prior to the washing process. These components are susceptible to mechanical stress from the cleaning mechanisms, potentially resulting in structural damage or detachment. Consequently, this risk remains a critical factor limiting the reliability and broader user acceptance of automatic car wash systems.

To fundamentally prevent physical damage during automatic car washing, it is essential to develop a system that detects surface attachments on the vehicle and adaptively controls the washing equipment. However, accurate detection is challenging because these attachments are small (e.g., antennas), possess irregular geometries due to aftermarket modifications, and exhibit high diversity without standardization. Conventional commercial systems relying on ultrasonic sensors [5] or classical computer vision algorithms [6,7] lack the robustness required to reliably identify objects with such variable shapes. In contrast, deep-learning-based object detection methods [8,9] have demonstrated the ability to effectively capture complex visual patterns by learning representations from training data. Therefore, robust detection of attachments with varying scales and irregular shapes requires deep learning approaches trained on domain-specific data that reflect the visual characteristics of car wash environments.

In real-world car wash environments, variations in camera installation height, viewing angles, lighting conditions, and backgrounds lead to significant divergence in the appearance distribution of identical attachments. The existing convolutional neural network (CNN)-based detectors primarily rely on local visual features; a consequently, their performance often degrades when encountering instances with viewpoints, scales, or shapes not represented in the training data. However, while changes in camera perspective alter object appearance, the relative spatial relationships and class co-occurrence patterns remain consistent due to the inherent functional structure of vehicles. For instance, roof carriers and taxi signs are generally located at the center of the roof. Furthermore, semantic correlations persist across varying capture conditions; for example, emblems typically co-occur with front wipers and side mirrors. Therefore, to achieve robust performance under diverse imaging conditions, it is necessary to adopt an approach that exploits these invariant spatial constraints and semantic relationships alongside visual features.

Along this direction, RelationNet [10] and SGRN [11] have been proposed to model spatial and semantic relationships between objects. However, these methods learn relational structures from large-scale general datasets with abundant training samples. When applied to domain-specific scenarios with scarce training data, such data-driven relational learning become less reliable.

To address this issue, we propose a novel framework that integrates a YOLOv11-based [12] object detector with a graph neural network (GNN) for relationship inference. We introduce a spatial graph module designed to capture invariant spatial features, which enhances feature representations to distinguish between morphologically distinct attachments located in similar spatial regions. Furthermore, we incorporate a class graph module to model semantic correlations between attachment categories, thereby improving classification accuracy for ambiguous objects. These dual graph modules are integrated directly into a YOLO pipeline at two distinct levels of the detection process. Unlike prior methods, our framework employs a domain-informed prior adjacency matrix rather than learning adjacency from data. Experimental results on a real-world car wash dataset demonstrate that the proposed method achieves superior performance compared to the baseline YOLOv11. In particular, the ablation study confirms that the prior adjacency matrix outperforms the learned alternative under limited training samples. Notably, our approach yields significant improvements in detecting challenging categories, such as emblems and carriers, which previous models struggled to identify accurately. In summary, the main contributions of this paper are as follows:

- We propose a robust vehicle attachment detection framework tailored for automated car washes to reliably identify diverse exterior components and prevent physical damage.
- We introduce a custom, real-world dataset collected from operating car washes, providing a practical benchmark with diverse viewpoints and realistic backgrounds.
- We propose dual graph modules capturing invariant spatial constraints and semantic correlations, which significantly improve the detection performance of ambiguous objects like emblems and carriers.

The remainder of this paper is organized as follows. Section 2 reviews related work on vehicle part detection and vision graph networks. Section 3 details the proposed detection framework, explaining the formulations of the spatial graph module and the class graph module. Section 4 describes the custom dataset construction and presents comprehensive experimental results, including comparative evaluations and ablation studies. Finally, Section 5 concludes the paper.

## 2. Related Work

### 2.1. Car Part Detection

Deep-learning-based object detection has become a core component in autonomous driving, with substantial progress in both accuracy and efficiency. Early work in this area largely relied on CNN detectors, where the YOLO series [13,14,15] is widely adopted for real-time vehicle bounding-box detection due to its favorable computational efficiency–accuracy trade-off. More recently, the introduction of vision transformer (ViT) [16] has accelerated the development of transformer-based detectors such as DETR [17,18], which aim to leverage global image context through attention mechanisms. Despite these advances, the existing studies primarily target vehicle-level perception in road-driving scenarios—detecting the overall vehicle silhouette or recognizing fixed components [19,20] for vehicle type identification. This focus leaves a gap for settings where the safety-critical objects are not the vehicle itself but small, irregular, and highly variable protruding attachments on the vehicle exterior. In automatic car wash environments, such attachments are typically small, can exhibit user-dependent and irregular shapes due to tuning, and span a wide variety. Consequently, prior vehicle detection paradigms are not directly aligned with the requirements of precisely detecting atypical exterior attachments that are prone to damage in the specialized domain of automatic car wash processes. This work thus represents one of the first systematic investigations into vision-based vehicle exterior attachment detection tailored to the automatic car wash domain.

### 2.2. Vision Graph Network

Convolutional neural networks are effective at extracting local features, but their fixed receptive fields can limit the modeling of global information. To address this limitation, RelationNet [10] proposes an object relation module that processes detected objects jointly by modeling interactions between appearance features and geometric information through an attention mechanism. While ViT also addresses the limited receptive field of CNNs by capturing global context through self-attention, it incurs excessive computational complexity. To bridge this trade-off, GNNs [21,22] have been introduced in the vision domain as a way to model relational structure. Vision GNN (ViG) [23] represents an image as a flexible graph and learns relational information among nodes. To mitigate the high complexity associated with ViT, ViG constructs edges using the K-Nearest Neighbor (KNN) algorithm [24], connecting only semantically related nodes, which enables global-context processing with linear computational complexity. However, early ViG designs can be limited in capturing diverse object scales or dynamic shape variations when they rely on a fixed number of K neighbors or a static adjacency matrix. To solve these problems and maximize the efficiency of graph operations, research has been conducted to improve the adjacency matrix. MobileViG [25] introduces sparse vision graph attention (SVGA) and proposes a method to increase computational efficiency by introducing connections between nodes spaced at intervals of k from rows and columns based on image feature patches. However, MobileViG’s method of connecting only with fixed patterns still has limitations in dynamic feature extraction that considers the complex shape or semantic association of objects. GreedyViG [26] proposes an improved dynamic axial graph construction (DAGC) over the SVGA connection to overcome the limitations of fixed connections. In the existing SVGA relationship, the L1-distance between patches is calculated and L1>(μ−σ) is set as a threshold. The graph is then dynamically updated to drop edges between patches whose distance exceeds the threshold.

In addition to image classification backbones, GNNs have also been applied to enhance feature representations in object detection. SGRN [11] constructs a sparse graph over region proposals using their visual features. It then uses learnable Gaussian kernels to model pairwise spatial information during graph reasoning. GraphFPN [27] introduces a graph pyramid network that inherits its topology from superpixel hierarchies of the input image. It enables simultaneous feature interactions across all scales through contextual and hierarchical graph layers. DGFD [28] proposes a dual-graph convolutional network for infrared and visible image fusion. A contextual graph module aggregates features at multiple levels and a content graph module fuses features across modalities for object detection under low light conditions. These works confirm that modeling graph relationships on image features is effective for object detection.

## 3. Methods

**Figure 2:**
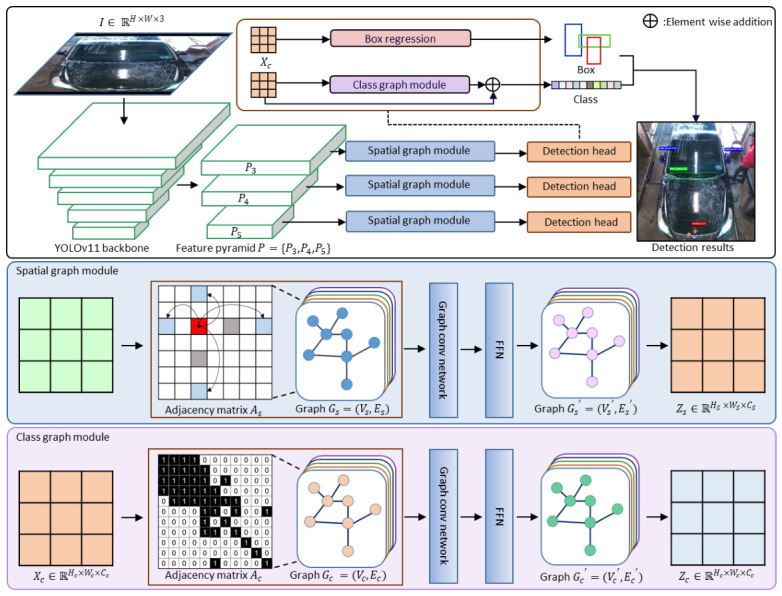
The overall architecture of the proposed graph YOLOv11 framework.

Our system builds upon the YOLOv11 architecture and introduces an enhanced framework that integrates dual graph modules to capture visual relationships specific to the car wash environment. As illustrated in Figure 2, the overall detection system comprises a YOLOv11-based backbone, a detection head, a spatial graph module, and a class graph module. Given an input image $I\in R^{H\times W\times 3}$, the backbone extracts multi-scale feature maps denoted as $P=\{P_{3},P_{4},P_{5}\}$. These feature maps are processed in parallel by the spatial graph module and the detection head. The spatial graph module represents a feature map $X_{s}\in R^{H_{S}\times W_{S}\times C_{S}}$ at the patch level as a graph $G_{s}=\left(V_{s},E_{s}\right)$. Here, $V_{s}$ and $E_{s}$ denote the node and edge sets, respectively. Concurrently, the class graph module is coupled with the classification branch of the detection head. It models semantic relationships among objects by treating the predicted class logits as nodes. Finally, the features refined by both graph modules are fused to predict the localization and classification of each object.

### 3.1. Graph Construction

In this study, we process the feature maps from the backbone by converting them into a graph structure, adopting the graph construction method of Vision GNN. The feature map $X\in R^{H\times W\times C}$ is divided into *N* distinct patches. These patches correspond to the vertices of the graph. Specifically, each node $v_{i}$ represents the visual information contained within the *i*-th patch. Each patch is transformed into a *D*-dimensional feature vector $x_{i}$, resulting in a feature set $X=\{x_{1},x_{2},\ldots ,x_{N}\}$, where i=1,2,…,N. For each node $v_{i}$, we establish directed edges connecting it to its neighbors $v_{j}$. Let *E* denote the set of all edges. The input image features are thus converted into a graph G=(V,E), which serves as the input to the graph network. We apply this graph mapping process across two distinct domains that differ in node semantics. First, the spatial graph module defines patches of the feature map as nodes. This design preserves the spatial localization of objects. Second, the class graph module defines the object classes as nodes. This allows the model to learn semantic interrelationships between different object categories. Consequently, our system utilizes both invariant spatial information and semantic relationships through these dual graph modules.

### 3.2. Spatial Graph Module

This module is designed to capture the geometric features of attachments with diverse shapes such as roof carriers and rear tires within the car wash environment. First, the input feature map $X_{s}\in R^{H_{S}\times W_{S}\times C_{S}}$ is partitioned into *N* patches. The resulting flattened feature vectors $X_{s}\in R^{N\times D}$ are defined as the node set $V_{s}$. These *N* nodes maintain a 2D spatial grid structure to preserve local geometry for shifting operations. GreedyViG employs DAGC primarily as an integrated convolution block. In contrast, our method decouples this process. We explicitly utilize the DAGC strategy as an adjacency construction mechanism to define the adjacency matrix $A_{s}$. The *N* nodes are inherently indexed by their fixed coordinates within the spatial grid.

Algorithm 1 details the explicit edge construction process.
**Algorithm 1:** Dynamic Edge Construction and GCN Node Update**Input:** Feature map $X_{s}\in R^{H_{S}\times W_{S}\times C_{S}}$, Shift *K***Data:** Patch Nodes $X_{s}\in R^{N\times D}$ where $N=H_{S}\times W_{S}$; Learnable Weight $W_{out}$;           Adjacency $A_{s}$ where $A_{s}\left[i,j\right]=1\Leftrightarrow e_{ij}\in E_{s}$;**Output:** Updated Nodes $Y_{s}$// 1. Global Patch Distance Setup (Threshold τ)$X_{flip}\leftarrow \mathrm{FlipQuadrants}\left(X_{s}\right)$;$D_{global}\leftarrow {∥X_{s}-X_{flip}∥}_{2}$;$\tau \leftarrow \mathrm{Mean}\left(D_{global}\right)-\mathrm{Std}\left(D_{global}\right)$;// 2. Dynamic Adjacency Construction**for** *axis* ∈{Vertical,Horizontal} **do**       m←1;       **while** m·K<Size(axis) **do**           $X_{nb}\leftarrow \mathrm{Roll}\left(X_{s},\mathrm{axis},m\cdot K\right)$;           $dist\leftarrow ∥X_{s}-X_{nb}{∥}_{2}$;           **if** dist<τ **then**               $e_{ij}\leftarrow 1$;                                                  //Edge exists ($e_{ij}\in E_{s}$)           **else**               $e_{ij}\leftarrow 0$;                                                                             //No edge           **end**           m←m+1;       **end****end**// 3. Graph Node Update via GCN$Y_{s}\leftarrow \sigma \left(\mathrm{GraphConv}\left(X_{s},\;A_{s}\right)\;W_{out}\right)+X_{s}$;**return** $Y_{s}$;

To establish the dynamic threshold τ for edge pruning, we first analyze the distance distribution among the *N* patch nodes. Calculating all $O(N^{2})$ pairwise patch distances is computationally expensive. Therefore, we estimate the global distribution by comparing the original patches with their spatially flipped counterparts. Here, FlipQuadrants(·) denotes a spatial quadrant flip operation that rearranges patch positions to approximate global pairwise distances efficiently. Subsequently, we explore candidate neighboring patches along rows and columns at intervals of *K*. We calculate the Euclidean distance between these corresponding patch vectors. An edge is established by setting $A_{s}\left[i,j\right]=1$ if the distance falls below the dynamically computed threshold τ. This pruning ensures that only semantically related patches remain connected, eliminating connections with low information relevance. After constructing the dynamic adjacency $A_{s}$, we update the node features using a graph convolution network (GCN) operation. This update is formulated as:

(1) $$Y_{s}=\sigma \left(\mathrm{GraphConv}\left(X_{s},A_{s}\right)W_{\mathrm{out}}\right)+X_{s}$$

Here, $Y_{s}$ denotes the updated features. The function σ represents the gaussian error linear unit (GELU) activation function. The matrix $W_{\mathrm{out}}$ is the learnable weight matrix. The GraphConv function acts strictly as a message-passing step over the defined edges $A_{s}$. Subsequently, we apply a feed-forward network (FFN) module formulated as:

(2) $$Z_{s}=\sigma \left(Y_{s}W_{1}\right)W_{2}+Y_{s}$$

In this equation, $Z_{s}$ represents the final output features. The matrices $W_{1}$ and $W_{2}$ denote the weights of the fully connected layers. By aggregating information exclusively through the dynamically constructed edges between highly correlated patches, this process enhances critical shape representations and filters out unnecessary noise.

### 3.3. Class Graph Module

**Figure 3:**
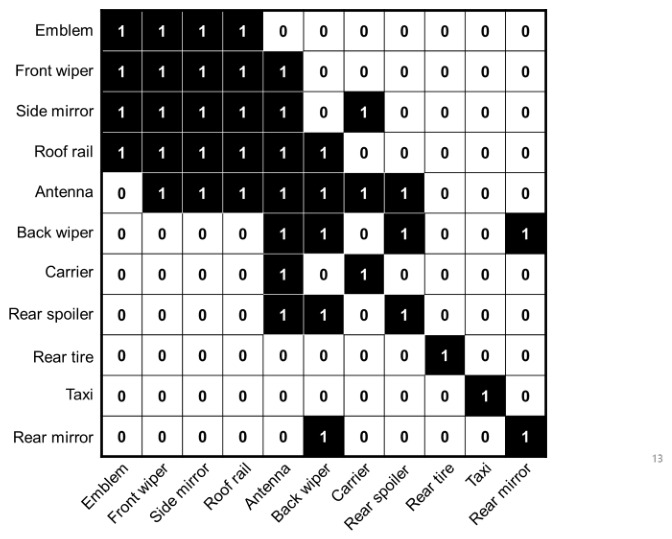
Visualization of the defined adjacency matrix of the relationships between vehicle attachment classes. Here, 1 indicates a co-occurring pair and 0 indicates no co-occurrence.

This module infers the relationships between objects to enhance the detection reliability of objects that are ambiguous or occluded by visual information alone. The input feature map $X_{c}\in R^{H_{c}\times W_{c}\times C_{c}}$ of the Detection Head is divided into *N* patches, resulting in $X_{c}\in R^{N\times D}$. The feature vector $X_{c}\in R^{N\times D}$ is defined as a node $V_{c}$. The class score output from the class branch is also treated as a node $V_{c}$, where each node represents the score indicating the presence of a specific class at that location. The edge $E_{c}$ between nodes is represented as an adjacency matrix $A_{c}$, capturing the semantic correlation between classes, as shown in the following Figure 3. The adjacency matrix $A_{c}$ is constructed based on domain knowledge of viewpoint-dependent attachment co-occurrence in the car wash environment. The overhead camera at the car wash entrance captures vehicles from three distinct viewpoint regions: front, top, and rear. Attachments that consistently co-occur within the same viewpoint region are connected ($A_{c}\left[i,j\right]=1$), while those belonging to different regions are not ($A_{c}\left[i,j\right]=0$). For instance, the emblem, front wiper, side mirror, and roof rail are mutually connected as front-region attachments, whereas the back wiper, carrier, and rear spoiler form a rear-region group.

The update process is carried out through a GCN and a FFN, as described by the following equations:

(3) $$Y_{c}=\sigma \left(\mathrm{GraphConv}\left(X_{c}W_{\mathrm{in}}\right)W_{\mathrm{out}}+X_{c}\right)$$

(4) $$Z_{c}=\sigma \left(Y_{c}W_{1}\right)W_{2}+Y_{c}$$

Each class node updates its state by referencing information from neighboring class nodes, using the adjacency matrix and learnable weights. Finally, the feature map $Z_{c}\in R^{H_{c}\times W_{c}\times C_{c}}$, inferred through the graph, is added to the original logit via a residual connection. This process improves the class accuracy of the detection head by leveraging semantic relationships between classes without compromising the visual features.

## 4. Experiments

### 4.1. Dataset Construction and Preprocessing

**Figure 4:**
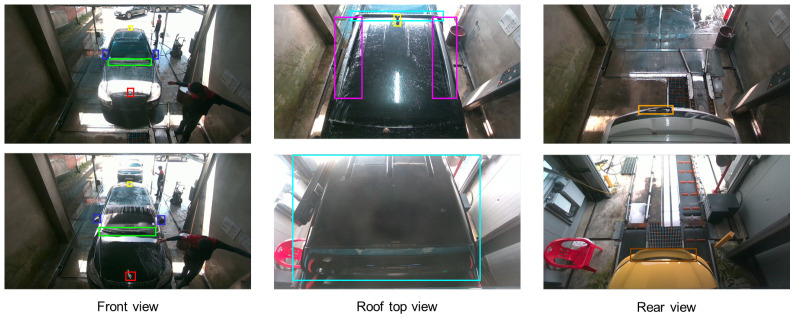
Visual examples from the automatic car wash environment dataset, demonstrating the appearance variations of exterior attachments across front, roof, and rear viewpoints. Each color box represents a distinct object class and boxes of the same color indicate the same class.

**Figure 5:**
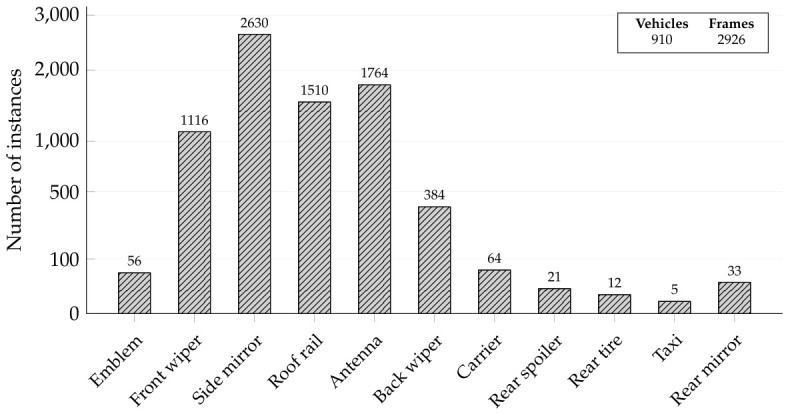
Comprehensive overview of the custom car wash dataset. The bar chart shows the total number of instances for each attachment category, while the inset summarizes the counts of vehicles, frames, and all classes.

In this study, to verify the performance of attachment detection even when the viewpoint changes, we built our own dataset using entry detection cameras installed in domestic automatic car washes. Figure 1b illustrates the overall data collection environment, including the deployed camera system and the specific hardware configuration. The data collection system is based on the NVIDIA Jetson AGX Orin device (NVIDIA Corporation, Santa Clara, CA, USA) and the Intel RealSense D455i camera (Intel Corporation, Santa Clara, CA, USA). The camera device was installed at the top center of the car wash entrance and captured the front of the entering vehicle. To reflect domain deviations according to camera installation angles, data were collected at two angles, 37 degrees and 57 degrees, respectively, relative to the ground. The initial data collection targeted a total of 910 vehicles in a real-world car wash environment. The entering vehicles were recorded as continuous video sequences at a resolution of 1080×720 with a frame rate of 15 frames per second. To ensure the quality and reliability of the dataset, a rigorous data preprocessing pipeline was applied to these video streams. First, redundant frames were systematically filtered out. Subsequently, we carefully curated a final high-quality dataset comprising 2926 distinct images, selecting only the frames where the vehicles and their exterior attachments were clearly visible. Figure 4 presents representative samples from this curated dataset, illustrating the diverse vehicle types and varying camera perspectives. Finally, to ensure a fair evaluation under limited data availability, we performed a stratified 8:2 train–test split, so that the class distribution was preserved in both the training and test sets. Figure 5 summarizes the detailed statistics of the constructed dataset, outlining the total volume of vehicles and extracted frames alongside the specific frequency of each attachment instance.

### 4.2. Experimental Environment and Evaluation Metrics

The model implementation and performance evaluation of this study were performed in a workstation environment equipped with an Intel i9-10900K CPU, 64GB RAM, and NVIDIA GeForce RTX 3090 GPU. We constructed a custom dataset specifically for the car wash entrance environment described in Section 4.1 to facilitate training and evaluation. For training and inference of the proposed model, the PyTorch 2.5.1 framework and CUDA 12.1 library were used in a Python 3.11 environment. Batch size and learning rate, which are hyperparameters for model optimization, were set to 8 and 0.0005. The entire training was conducted for a total of 100 epochs, and warmup scheduling was applied for the first 10 epochs to alleviate instability in the early stage of training.

In this study, we use Precision, Recall, and mean average precision (mAP), which are standard evaluation indices in the field of object detection, to quantitatively verify the object detection performance of the proposed model. Precision refers to the proportion of true positives among the results detected by the model, and indicates how much false positives are minimized. Recall is the percentage of ground truth data that the model successfully predicts, indicating how completely the model captures the relevant targets. The area under the Precision–Recall curve showing the relationship between these two indicators is defined as average precision. Intersection over union (IoU) is the area of the intersection between the bounding box predicted by the model and the actual correct box divided by the area of the union of the two boxes, and is an indicator of how closely the two boxes match. mAP50 is the mAP when the IoU threshold is set to 0.5, and is a method of considering a correct answer when the predicted box and the correct answer box overlap by more than 50%. mAP50-95 is the average value of APs measured while increasing the IoU threshold from 0.5 to 0.95 in steps of 0.05.

### 4.3. Quantitative Results

In this study, to verify the objective performance of the proposed technique, comparative experiments were conducted with models widely used in the field of object detection. The object detection models selected include the base model YOLOv11, YOLOv8, and the latest transformer-based detection models RT-DETR [29] and D-FINE [30]. The experimental results are summarized in Table 1, and the quantitative evaluation indices of Precision, Recall, mAP50, and mAP50-95 were used for each model.

Experimental results show that the transformer-based RT-DETR and D-FINE achieve mAP50 of 96.1% and 96.5% respectively, remaining competitive with the baseline YOLOv11’s 96.3%. However, the proposed model achieves an mAP50 of 97.9% surpassing the latest transformer-based model D-FINE which records 96.5% by 1.4%. Furthermore, the proposed model records the highest Recall of 93.8% among all compared methods. These results confirm that combining spatial and semantic graph relationship information with CNN-based feature extraction is more effective in specialized environments such as car wash entrances.

Table 2 summarizes the computational cost and inference speed of evaluated models. FPS and inference time were measured on an NVIDIA Jetson Orin Nano board to reflect practical deployment conditions. YOLOv8-s, YOLOv11-s, and D-FINE-s are relatively lightweight models with parameters ranging from 9.4 M to 11.3 M. The proposed model has 32 M parameters and 40.7 GFLOPs due to the additional GCN operations introduced by the dual graph modules. However on the embedded board the proposed model achieves 22.5 FPS with 44.5 ms inference time and outperforms D-FINE-s which records 12.7 FPS with 78.5 ms. This suggests that the combination of CNN and GCN is more efficient than transformer-based architectures for real-time inference on embedded devices. With an inference speed of 22.5 FPS on the embedded board, the proposed model provides low latency for real-time operation. Therefore, this performance supports the practical deployment of the proposed model in real-world car wash entrance environments.

In this study, we performed a detailed analysis of the detection ability for each class, as well as the overall performance across all objects. Table 3 presents the detection performance metrics for each class. The results demonstrate high detection performance across most classes with consistent improvements in both mAP50 and mAP50-95. The most notable gains are observed in Emblem and Carrier which are the two classes that the baseline model struggled with most due to small object size and high intra-class shape diversity. For Emblem, the small-scale model improves both mAP50 and mAP50-95 by 6.4% and 10.7%, respectively, while the nano-scale model yields an mAP50-95 improvement of 8.7%. For Carrier, the nano-scale model achieves improvements in both mAP50 and mAP50-95 by 10.1% and 20.1%, respectively, while the small-scale model yields an mAP50 improvement of 5.1%. These improvements are attributed to the dual graph modules which encode invariant spatial positions of attachments and semantic co-occurrence patterns among classes thereby enabling more reliable detection of visually ambiguous objects.

### 4.4. Qualitative Results

**Figure 6:**
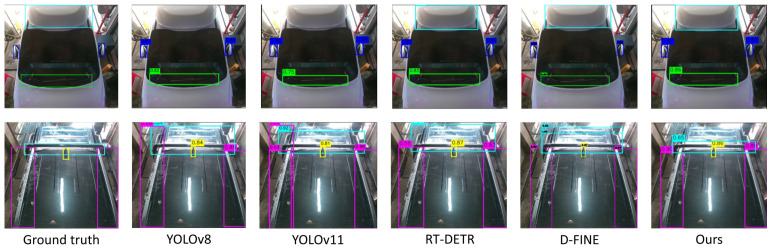
Qualitative comparison of object detection results. Each color box represents a distinct object class and boxes of the same color indicate the same class.

Figure 6 presents the visualization results comparing the baseline and our proposed method. Notably, for the Carrier class, which exhibits high intra-class variance and limited training samples, the results clearly demonstrate the superiority of the proposed model. In the initial example, the target object is difficult to recognize due to its subtle visual appearance, and the baseline YOLO models fail to detect it. In contrast, both the state-of-the-art transformer-based models (RT-DETR, D-FINE) and our proposed model successfully capture the object, demonstrating robust detection capabilities even under the dual challenges of data scarcity and complex backgrounds. Second, in the subsequent case comparing bounding box accuracy, the proposed model exhibits precise localization performance. While both the YOLO baselines and the transformer models tend to overestimate the object area compared to the ground truth (GT), our model generates a tight bounding box that aligns most closely with the GT. This suggests that the proposed graph module effectively learns invariant spatial features from sparse data, thereby significantly improving localization accuracy.

### 4.5. Ablation Experiment

We conducted an ablation study to analyze the individual contributions of the spatial graph module and the class graph module to the proposed framework. Using YOLOv11 as the baseline, we evaluated performance changes by incrementally integrating each module. Table 4 summarizes the quantitative results for each configuration. First, categories such as Carriers exhibit significant intra-class variance in appearance, making them challenging to distinguish based solely on visual features. When the spatial graph module was individually applied to the baseline, the nano-scale and small-scale models achieved mAP50 improvements of 0.5% and 0.9%, respectively. This suggests that the spatial graph module effectively captures the spatial characteristics of vehicle attachments within the car wash environment. We attribute this improvement to the module’s ability to encode spatial priors, such as fixed camera angles and the relative positions of attachments, which enhances object localization accuracy.

Additionally, applying the class graph module in isolation also yielded performance gains. Experimental results indicate slight mAP50 improvements for both the nano- and small models compared to the baseline. In particular, modeling inter-class semantic relationships improved detection for visually ambiguous objects, such as Emblems. To validate the design of each module, we conducted additional experiments. Table 5 shows the detection performance of the Ours-s model under different shift parameter *K* values in the spatial graph module. The parameter *K* controls the interval at which candidate neighboring patches are explored during dynamic adjacency construction. The results show that K=2 achieves the highest mAP50 of 97.9%, while performance degrades at larger *K* values, dropping to 97.4% at K=4 and sharply declining in mAP50 from 97.9% to 97.3% at K≥8. This indicates that smaller intervals preserve important local spatial dependencies, whereas overly sparse graphs lose neighborhood information. Table 6 compares the prior adjacency matrix against a learned variant in the class graph module. The learned variant initializes the adjacency matrix randomly and optimizes it jointly with the model parameters during training. Across both model scales, the prior adjacency matrix consistently achieves higher than the learned alternative. The Ours-s model with the prior matrix achieves an mAP50 of 97.9% compared to 96.5% for the learned variant, and the Ours-n model improves from 97.1% to 95.8%. These results suggest that the prior matrix encodes domain knowledge that is difficult to learn from limited training data. The proposed framework integrating both modules achieved the highest performance among all comparative configurations. This demonstrates that synergizing the invariant spatial features from the spatial graph module with the semantic relationship cues from the class graph module leads to robust detection performance.

## 5. Conclusions

In this paper, we proposed a novel object detection framework based on YOLOv11 to prevent damage to vehicle attachments during automatic car washing. We validated the efficacy of our method using a dataset collected in real-world car wash environments. To the best of our knowledge, this work represents one of the first systematic investigations into vision-based detection of vehicle exterior attachments tailored to the automatic car wash domain. The proposed model achieved a high mAP50 of 97.9%, outperforming state-of-the-art detectors such as D-FINE (96.5%) and RT-DETR (96.1%). These results demonstrate that the proposed dual graph modules effectively capture the spatial and semantic relationships between objects. By integrating relational inference with GNNs, our approach enables robust attachment detection even under diverse camera viewpoints and background conditions. Consequently, this work is expected to significantly enhance the safety and reliability of automatic car wash systems.

Despite these promising results, this study has certain limitations. First, the dataset collected in the car wash environment contains an insufficient number of samples for specific attachments, potentially limiting the model’s generalizability. Second, the current class graph module relies on fixed relationships between attachment classes, which may vary under different real-world contexts. Therefore, future research will focus on expanding the dataset with a wider variety of attachment samples to ensure robust generalization. Additionally, we plan to explore a more adaptive graph design capable of generalizing inter-class relationships, aiming to further enhance detection robustness across diverse vehicle types and car wash environments.

## Figures and Tables

**Table 1:** Quantitative comparison with recent YOLO-series and transformer-based models. Best results are shown in bold and second-best results are underlined.

Method	Params	Precision	Recall	mAP50	mAP50-95
YOLOv8-s [15]	11.3 M	0.880	0.913	0.954	0.646
YOLOv11-s [12]	9.4 M	0.887	0.875	0.963	0.641
RT-DETR-HGNetv2-L [29]	32 M	0.895	0.934	0.961	0.685
D-FINE-s [30]	10 M	**0.938**	0.936	0.965	0.675
Ours	32 M	0.910	**0.938**	**0.979**	**0.699**

**Table 2:** Computational cost comparison of compared models.

Method	Params	GFLOPs	FPS	Inference Time (ms)
YOLOv8-s [15]	11.3 M	10.6	30.2	28.5
YOLOv11-s [12]	9.4 M	21.3	33.1	30.2
RT-DETR-HGNetv2-L [29]	32 M	103.5	10.6	94.3
D-FINE-s [30]	10 M	25.2	12.7	78.5
Ours	32 M	40.7	22.5	44.5

**Table 3:** Comparison of class-wise detection performance between the baseline YOLOv11 and the proposed method across different model scales.

Class	YOLOv11-n		Ours-n		YOLOv11-s		Ours-s	
	mAP50	mAP50-95	mAP50	mAP50-95	mAP50	mAP50-95	mAP50	mAP50-95
Emblem	0.934	0.375	0.935 (+0.001)	0.462 (+0.087)	0.924	0.390	0.988 (+0.064)	0.497 (+0.107)
Front wiper	0.967	0.642	0.989 (+0.022)	0.671 (+0.029)	0.969	0.653	0.981 (+0.012)	0.692 (+0.039)
Side mirror	0.994	0.724	0.995 (+0.001)	0.732 (+0.008)	0.992	0.734	0.993 (+0.001)	0.747 (+0.013)
Roof rail	0.957	0.767	0.955 (−0.002)	0.800 (+0.033)	0.962	0.793	0.947 (−0.015)	0.798 (+0.005)
Antenna	0.975	0.608	0.973 (−0.002)	0.609 (+0.001)	0.980	0.628	0.982 (+0.002)	0.638 (+0.010)
Back wiper	0.968	0.481	0.948 (−0.020)	0.513 (+0.032)	0.958	0.528	0.977 (+0.019)	0.574 (+0.046)
Carrier	0.803	0.524	0.904 (+0.101)	0.725 (+0.201)	0.871	0.571	0.922 (+0.051)	0.689 (+0.118)
Rear spoiler	0.995	0.709	0.995 ( - )	0.899 (+0.190)	0.995	0.691	0.995 ( - )	0.870 (+0.179)
Rear tire	0.995	0.697	0.995 ( - )	0.597 (−0.100)	0.995	0.699	0.995 ( - )	0.697 (−0.002)
Taxi	0.995	0.796	0.995 ( - )	0.895 (+0.099)	0.995	0.695	0.995 ( - )	0.796 (+0.101)
Rear mirror	0.972	0.662	0.995 (+0.023)	0.684 (+0.022)	0.955	0.676	0.995 (+0.040)	0.693 (+0.017)
Average	0.960	0.635	0.971 (+0.011)	0.690 (+0.055)	0.963	0.642	0.979 (+0.016)	0.699 (+0.058)

Positive changes are highlighted in ForestGreen and negative changes in gray.

**Table 4:** Ablation study on the effectiveness of the spatial and class graph modules across different model scales. Best results are shown in bold.

Model	Spatial Module	Class Module	Precision	Recall	mAP50	mAP50-95
YOLOv11-n			**0.898**	0.913	0.960	0.635
	✓		0.884	0.911	0.965	**0.692**
		✓	0.890	0.900	0.960	0.685
	✓	✓	0.875	**0.937**	**0.971**	0.690
YOLOv11-s			0.887	0.875	0.963	0.641
	✓		0.840	0.912	0.972	**0.702**
		✓	**0.928**	0.928	0.966	0.692
	✓	✓	0.910	**0.938**	**0.979**	0.699

**Table 5:** Ablation study of the shift parameter *K* in the spatial graph module using the Ours-s model. Best results are shown in bold.

Model	K	Precision	Recall	mAP50	mAP50-95
Ours-s	2	0.910	**0.938**	**0.979**	**0.699**
	4	**0.917**	0.912	0.974	0.686
	8	0.908	0.910	0.973	0.689
	12	0.908	0.910	0.973	0.689

**Table 6:** Ablation study comparing adaptive and prior adjacency matrix in the class graph module. Best results are shown in bold.

Model	Adjacency Matrix	Precision	Recall	mAP50	mAP50-95
Ours-n	Adaptive	**0.901**	0.884	0.958	0.641
	Prior	0.875	**0.937**	**0.971**	**0.690**
Ours-s	Adaptive	**0.917**	0.896	0.965	0.660
	Prior	0.910	**0.938**	**0.979**	**0.699**

## Data Availability

The data presented in this study are available on request from the corresponding author.
